# iGUIDE: an improved pipeline for analyzing CRISPR cleavage specificity

**DOI:** 10.1186/s13059-019-1625-3

**Published:** 2019-01-17

**Authors:** Christopher L. Nobles, Shantan Reddy, January Salas-McKee, Xiaojun Liu, Carl H. June, J. Joseph Melenhorst, Megan M. Davis, Yangbing Zhao, Frederic D. Bushman

**Affiliations:** 1 Department of Microbiology, Perelman School of Medicine, University of Pennsylvania, 3610 Hamilton Walk, Philadelphia, PA 19104-6076 USA; 2 Center for Cellular Immunotherapies, Perelman School of Medicine, University of Pennsylvania, Philadelphia, PA USA; 3 Abramson Cancer Center, Perelman School of Medicine, University of Pennsylvania, Philadelphia, USA; 4 Pathology and Laboratory Medicine, Perelman School of Medicine, University of Pennsylvania, Philadelphia, USA; 5 Parker Institute for Cancer Immunotherapy, University of Pennsylvania, Philadelphia, USA

## Abstract

Genome engineering methods have advanced greatly with the development of programmable nucleases, but methods for quantifying on- and off-target cleavage sites and associated deletions remain nascent. Here, we report an improvement of the GUIDE-seq method, iGUIDE, which allows filtering of mispriming events to clarify the true cleavage signal. Using iGUIDE, we specify the locations of Cas9-guided cleavage for four guide RNAs, characterize associated deletions, and show that naturally occurring background DNA double-strand breaks are associated with open chromatin, gene dense regions, and chromosomal fragile sites. iGUIDE is available from https://github.com/cnobles/iGUIDE.

**Electronic supplementary material:**

The online version of this article (10.1186/s13059-019-1625-3) contains supplementary material, which is available to authorized users.

## Introduction

**Fig. 1:**
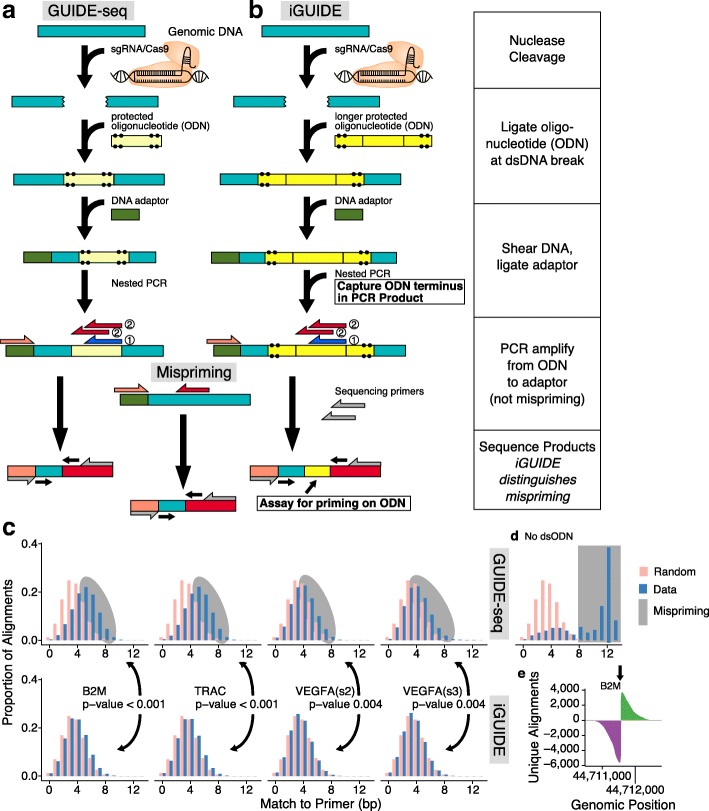
Diagram of the method, illustrating the strategy for improving specificity and examples of output. Procedure for GUIDE-seq (**a**) and iGUIDE (**b**). A dsODN is incorporated into DNA breaks. Amplification of flanking DNA, by nested-PCR, produces sequence copies indistinguishable from genomic mispriming when using the GUIDE-seq design. The modified dsODN of iGUIDE uses a reporter present in sequence output to identify correctly primed molecules. **c** Alignment of amplification primer and upstream sequence from uniquely identified sites in either GUIDE-seq or iGUIDE samples. We reasoned that amplification products resulting from mispriming should be just adjacent to sequences in the human genome with adventitious matches to the amplification primer sequence. Evidence for greater matching to primer sequences in a sample thus provides evidence for more mispriming. In the figure, the x-axis scores the match of the inferred human flanking DNA to the amplification primer (marked 2 in **a** and **b**); higher numbers of matching bases than seen for random sequences (light red) indicates probable mispriming. *P* values compare the distributions of the matches to the primer sequences in DNA samples detected for GUIDE-seq (top) and iGUIDE (bottom). **d** Sequence coverage of an on-target CRISPR site from iGUIDE data (gRNA targeting B2M)

Multiple methods have been developed for quantifying the distributions of DNA double-strand breaks in cells [1–15], which are important in tracking cleavage of designer nucleases used for gene modification in humans and many other purposes. All methods can be useful, and each has its own limitations and assumptions (Additional file 1: Table S1). Several methods label DNA double-strand breaks by recombination with an exogeneous marker DNA. AAV (AAV marking) [14], integration defective lentiviruses (IDLV marking) [10], and protected oligonucleotides (GUIDE-seq) [7] have all been used with success. GUIDE-seq (Fig. 1a) is particularly convenient, because it is simple to implement, and bypasses questions on the possible influence of innate immune sensing of viral proteins when viral vectors are used for delivery. GUIDE-seq has been used widely, but as originally proposed, the method does not effectively filter out mispriming artifacts, leading us to propose an improvement which we named iGUIDE (Fig. 1b).

In the GUIDE-seq method (Fig. 1a), cells are exposed to designer nucleases such as Cas9/sgRNA complexes, and then a marker deoxyribonucleotide (ODN) is transfected into cells. Cellular DNA repair pathways then incorporate the oligonucleotide into the double-strand break site in the course of repair, thereby covalently marking the location of the break. Break sites can then be read out using ligation-mediated PCR (Fig. 1a, b), in which DNA is broken by sonication, adaptors are ligated to the broken DNA ends, then DNAs are amplified by two rounds of PCR using primers that bind to the adaptor and primers that bind to the incorporated ODN. PCR products are then analyzed by next-generation DNA sequencing and mapped onto the human genome scaffold.

However, a complication is that PCR primers will sometimes anneal to human DNA sequences other than the ODN and prime PCR. This results in PCR products that are indistinguishable from products formed by primer binding to the ODN, because the PCR primer contributes sequences identical to the ODN—thus mispriming will obscure the true distribution of cleavage sites. Estimates of Cas9/sgRNA off-target cleavage positions have varied widely, probably in part because of authentic variation among sgRNAs, but also likely due in part to variable admixture of mispriming artifacts.

Here, we present a revision of the GUIDE-seq method that allows mispriming artifacts to be distinguished from authentic ODN integration sites (Fig. 1b) and a vetted software pipeline to implement the analysis (available at https://github.com/cnobles/iGUIDE). The iGUIDE method involves the same steps as in GUIDE-seq, but a larger ODN is used (46 nt versus 34 nt). As a result, the PCR primer binding sites can be moved away from the junction between the ODN and flanking human-derived DNA, leaving a segment of the ODN in the final PCR product. Following DNA sequence acquisition, this ODN reporter sequence can be recognized in the DNA sequence data. Only products generated by PCR priming on the ODN will have this ODN reporter sequence—sequences acquired by mispriming will lack the ODN reporter. Thus, correct priming on the ODN can be distinguished from artifactual mispriming elsewhere in the human genome (Fig. 1b), a distinction that was not possible with the original GUIDE-seq design.

## Results and discussion

Given the ability to distinguish mispriming from correct priming, we investigated the frequency and distribution of mispriming events generated in model studies of sgRNA/Cas9 nuclease targeting four loci. The first two, in VEGFA, have been studied extensively previously and serve as methodological controls. The other two are in the beta2-microglobulin gene (B2M) and the T cell receptor alpha chain coding region (TRAC). The evaluation of these latter two targets is of interest for disrupting pathways of antigen detection, a crucial component in the development of allogenic T cell immunotherapies. Disruption of either T cell receptor alpha chain or beta chain is sufficient to knockout the T cell receptor, while B2M is essential for presentation of the HLA-I complex [16]. Samples were tested with or without the sgRNA and Cas9, and GUIDE-seq and iGUIDE were compared. All sgRNAs were tested in primary human T cells, which are of particular interest as substrates for modification due to their extensive use in human immunotherapy. Detailed protocols are provided in the “Methods” section and Additional file 2: Table S2 together with directions to custom software for sequence analysis.

Mispriming is readily detectable experimentally. In control reactions in which no ODN was added, amplification products were still detected, documenting amplification after binding of ODN primers to sequence-related sites on the human genome (Additional file 3: Table S3). An approach to quantifying mispriming in reactions with DNA from cells that were transfected with the ODN is shown in Fig. 1c. It is expected that mispriming takes place when PCR primers bind to human DNA sequences that happen to resemble the primer sequences—thus, inferred primer binding sites from mispriming events are expected to resemble the PCR primer sequence to a greater degree than is expected by chance. As can be seen, a substantial fraction of sites generated by GUIDE-seq (Fig. 1c, top) lacking the ODN reporter shows greater sequence homology to the PCR primer (blue) than random controls (red), indicative of widespread mispriming. However, samples where sites were filtered using resemblance to the iGUIDE dsODN reporter (Fig. 1c, bottom) were closer to the random control, indicating removal of misprimed sequences.

Figure 1d shows the sequence profile returned for on-target cleavage. The figure shows relative sequence coverage for the bases reported by iGUIDE surrounding the site of nuclease cleavage (arrow). The positions of the ODN also report the edges of deletions at the sites of sgRNA/CAS9 cleavage. Additional file 4: Figure S1 shows the data by site of DNA breakage.

A standard operating procedure for carrying out iGUIDE analysis is available in the Additional files (Additional file 5). We note that empirical experience shows that iGUIDE typically yields more total reads aligning to the human genome than does GUIDE-seq; possibly, the longer ODN is more stable in cells or incorporated by cellular enzymes more efficiently. In addition, we supply software that takes as input the raw iGUIDE sequence data and outputs a series of data tables and summaries. An example of such a reproducible report is in the Additional files (Additional file 6); while the most current version of the software is available here (https://github.com/cnobles/iGUIDE).

**Fig. 2:**
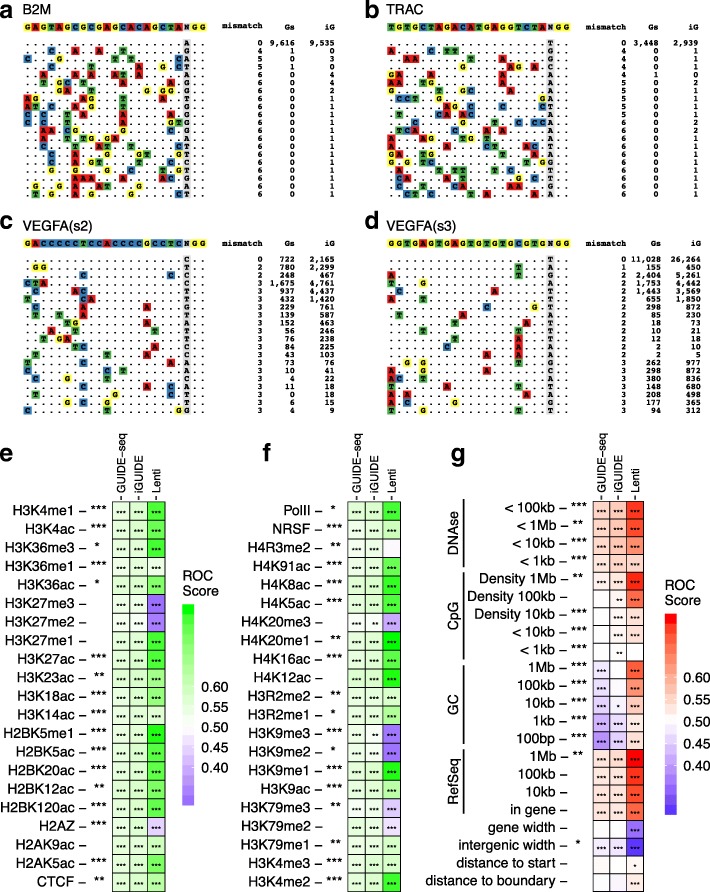
Distributions of DNA double-strand breaks in human cells analyzed by GUIDE-seq and iGUIDE. Sequences of suspected Cas9 edited sites associated with either the B2M (**a**), TRAC5 (**b**), or VEGFA guideRNAs (**c**, **d**). The number of guideRNA mismatches are annotated to the right of the associated sequence, as well as the number of inferred cells sampled, as reported by GUIDE-seq (Gs) or iGUIDE (iG) data. **e**–**g** Analysis of the distribution of spontaneous DNA double-strand breaks in cells relative to genomic annotation. Each column shows, from left to right, analysis of sites of dsDNA breaks inferred by iGUIDE and GUIDE-seq. The third column shows sites of lentiviral vector integration in T cells from Fraietta et al. [22] for comparison—HIV favors integration in active transcription units, which is reflected in the integration site preferences [23–25]. Rows summarize the relationship of each form of genomic annotation on the human genome to mapped sites. To generate the heat maps, sites are correlated with the density of genomic annotation in intervals along the genome, and co-occurrence summarized as receiver operating characteristic (ROC) curves. Positive associations (> 0.5) are shown by the higher values (red), negative associations (< 0.5) by the lower values (blue). No association (0.5) is shown white. Because the relevant widow size for comparison is unknown, multiple window sizes were tested. Asterisks on each tile compare the statistical significance for comparison to no association. * indicates 0.05 > *p* > 0.01; ** indicates 0.01 > *p* > 0.001; *** indicates *p* < 0.001. **e**, **f**: as in (**g**), but associations are shown relative to epigenetic marks mapped in T cells. In the analysis, 10 Kb chromosomal intervals were used for the comparison

One of the main applications of iGUIDE and GUIDE-seq is quantification of the specificity of cleavage, but the assumptions in the analysis strongly affect the outcome. Depending on assumptions, the proportion of on-target cleavage in our study ranged from 2.1 to 100% for the sgRNAs studied (Additional file 6). A complication is that DNA double-strand breaks are formed spontaneously during cell division at high rates in the absence of added nucleases—estimates range from ~ 10–50 per cell per cell cycle [17, 18]—resulting in a high background in assays of off-target cleavage. To account for this background, GUIDE-seq output has typically been filtered for a nearby match to sequences resembling the sgRNA binding site, and only those sites with some resemblance are scored as off-target cleavage. We thus analyzed our data requiring a match of 14/20 bases of the sgRNA recognition sequence and a perfect match to the protospacer adjacent motif to be present within 100 bases of the incorporation site. Using this filter, we found specificities ranging from 98.3 to 100% for B2M and TRAC sgRNAs and 2.2 to 29% for VEFGA sgRNAs. Without filtering by the match to the sgRNA, the estimates of percentage on-target were much lower, from 1.1 to 49%, likely due at least in part to the high frequency of spontaneous DNA breaks. Sequences at near matches to the sgRNA targets studied are shown in Fig. 2a–d; a diagram of the top 100 for each are in Additional file 7: Figure S2.

Improved filtering by iGUIDE allowed us to clarify the chromosomal features associated with spontaneous cellular DNA double-strand breaks and marking by ODN incorporation (Fig. 2e–g). Detailed analysis showed that spontaneous DNA double-strand breaks occur preferentially near active genes (Fig. 2g) and epigenetic marks associated with gene activity (Fig. 2e, f). Breaks also occur preferentially in AT-rich DNA and near previously annotated chromosomal fragile sites (13 to 19% enrichment, *p* value < 0.001 compared to random incorporation sites). The extent of these trends was obscured in GUIDE-seq data by admixture of mispriming artifacts. These findings now pose the question of whether chromatin structure and gene activity influence the initial formation of dsDNA breaks or the subsequent activity of repair pathways leading to ODN incorporation.

## Conclusions

In conclusion, iGUIDE provides a method for quantifying sites of nuclease cleavage free of confounding mispriming artifacts and allows more accurate assessment of the distribution of dsDNA breaks in cells.

## Methods

### Editing the genes encoding beta2-microglobulin and the T cell receptor alpha constant region with Cas9 in T cells

Cas9 protein was delivered complexed with a single-guide RNA (sgRNA) against B2M (guide RNA sequence: GAGTAGCGCGAGCACAGCTANGG), TRAC (guide RNA sequence: TGTGCTAGACATGAGGTCTANGG), VEGFA site 2 (guide RNA sequence: GACCCCCTCCACCCCGCCTCNGG), and VEGFA site 3 (guide RNA sequence: GGTGAGTGAGTGTGTGCGTGNGG). Primary human CD4+ and CD8+ T cells were isolated from healthy volunteer donors following leukapheresis by negative selection using RosetteSep Kits. Primary lymphocytes were stimulated with anti-CD3/CD28 beads for 3 days. The Cas9 guide RNA complex was formed by incubating (10 min) Cas9 protein at room temperature with guide RNA at a molar ratio of 1:2.4. On day 4, the Cas9 complexed with sgRNAs targeting B2M were electroporated into the cells. After expansion for an additional 6 days, the T cells were harvested and genomic DNA was isolated.

### Library preparation, DNA sequencing, and analysis

Libraries were prepared as described in the associated protocol for iGUIDE. Genomic DNA from samples was purified and randomly fragmented by ultrasonication. Adapters were ligated to end-repaired DNA, and targeted DNA was amplified through a nested-PCR from the incorporated dsODN to the ligated adapter sequence. Amplicons were purified and sequenced on an Illumina MiSeq with 300 cycle v2 reagent kits. Additional file 2: Table S2 presents oligonucleotides used in this study. Output sequence data was analyzed using the iGUIDE pipeline.

### iGUIDE standard operating procedure

An SOP for carrying out iGUIDE is associated with this manuscript (Additional file 5).

## Additional files

Additional file 1: **Table S1.** Methods for mapping sites of new DNA cleavage based on incorporation of new DNAs. (XLSX 10 kb)

Additional file 2: **Table S2.** Oligonucleotides used in this study. (XLSX 10 kb)

Additional file 3: **Table S3.** Evidence for mispriming: sequence reads recovered from control reactions containing DNA from T cells that were not treated with the double-stranded oligonucleotide (ODN). We note that additional mispriming events in cells that were transfected with the ODN may have a different character. Inspection of data suggests formation of chimeric PCR products, probably involving DNA chains amplified by priming on the ODN initially, which go on to form complex molecules that under some circumstances map in a fashion paralleling simple mispriming. The iGUIDE method filters out many of these more complex artifacts as well. (XLS 21 kb)

Additional file 4: **Figure S1.** Frequency of dsODN incorporation surrounding the expected Cas9 cleavage sites for B2M, TRAC5, and VEGFA sgRNAs. “Cells Observed” were quantified using lengths of flanking DNA fragments after sonication as a measure of independent isolation events. (PDF 169 kb)

Additional file 5: A Standard Operating Procedure for carrying out iGUIDE analysis. This report provides detailed protocols for carrying out the iGUIDE procedure. (PDF 107 kb)

Additional file 6: Automated iGUIDE Summary Report. This reproducible report includes specifics on the samples sequenced and annotated data for each, generated by a standardized software pipeline. (PDF 1035 kb)

Additional file 7: **Figure S2.** Human genome sequences with near matches to the B2M, TRAC5, and VEGFA sgRNAs, with data on frequency of incorporation of the dsODN. “Count” indicates the frequency of the sequence in the human genome; “mismatch” indicates the number of mismatches relative to the sgRNA recognition sequence; “Gs” indicates the number of isolations from GUIDE-seq; and “iG” indicates the number of isolations from iGUIDE. (PDF 3508 kb)
