# Supplementary figures and images for: iGUIDE: an improved pipeline for analyzing CRISPR cleavage specificity
Source: Genome Biol. 2019 Jan 17;20:14. doi: 10.1186/s13059-019-1625-3 (PMC6337799; doi:10.1186/s13059-019-1625-3)

**Figure S1**

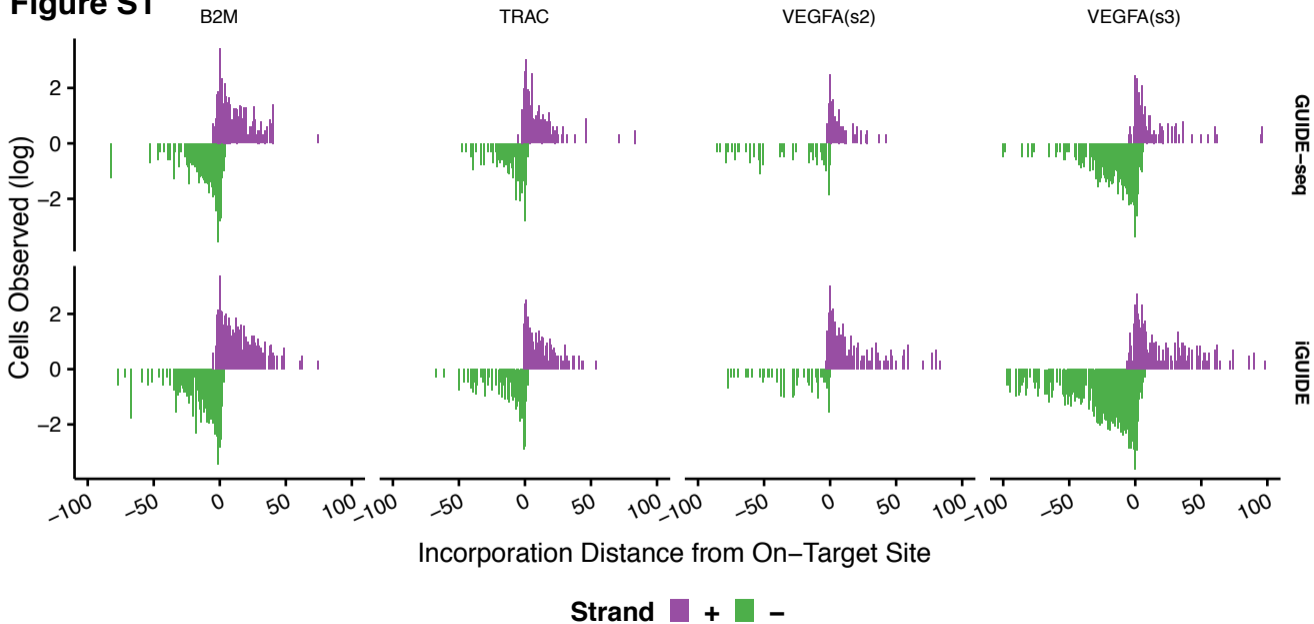

Supplement: Supplementary file 4 — Figure S1. Frequency of dsODN incorporation surrounding the expected Cas9 cleavage sites for B2M, TRAC5, and VEGFA sgRNAs. “Cells Observed” were quantified using lengths of flanking DNA fragments after sonication as a measure of independent isolation events. (PDF 169 kb) [file 13059_2019_1625_MOESM4_ESM.pdf]

Figure S2

A  
B2M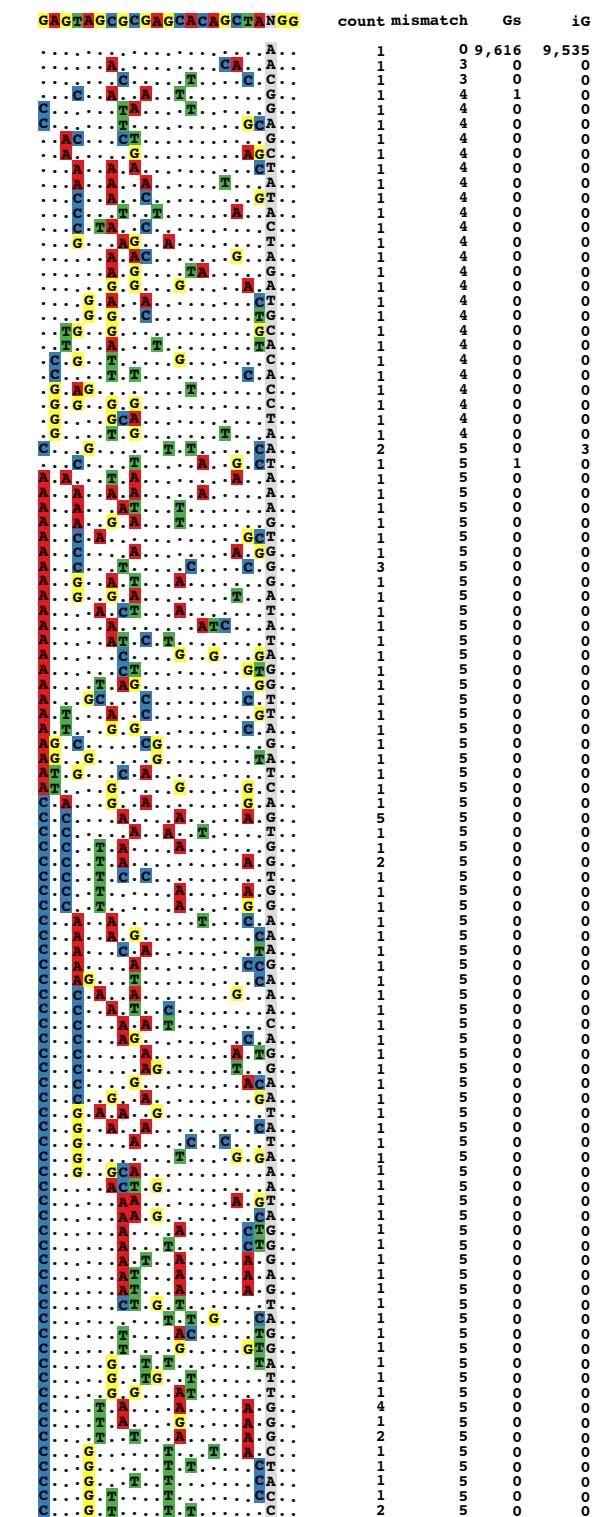B  
TRAC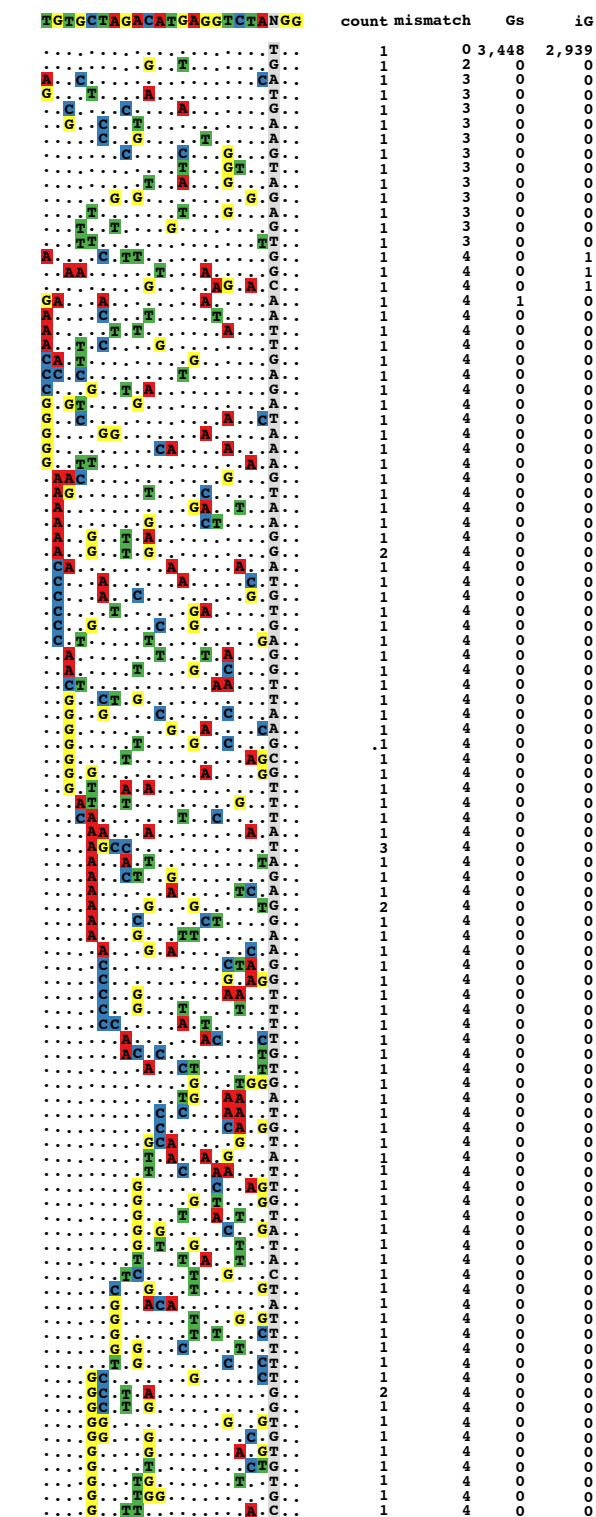C  
VEGFA(s2)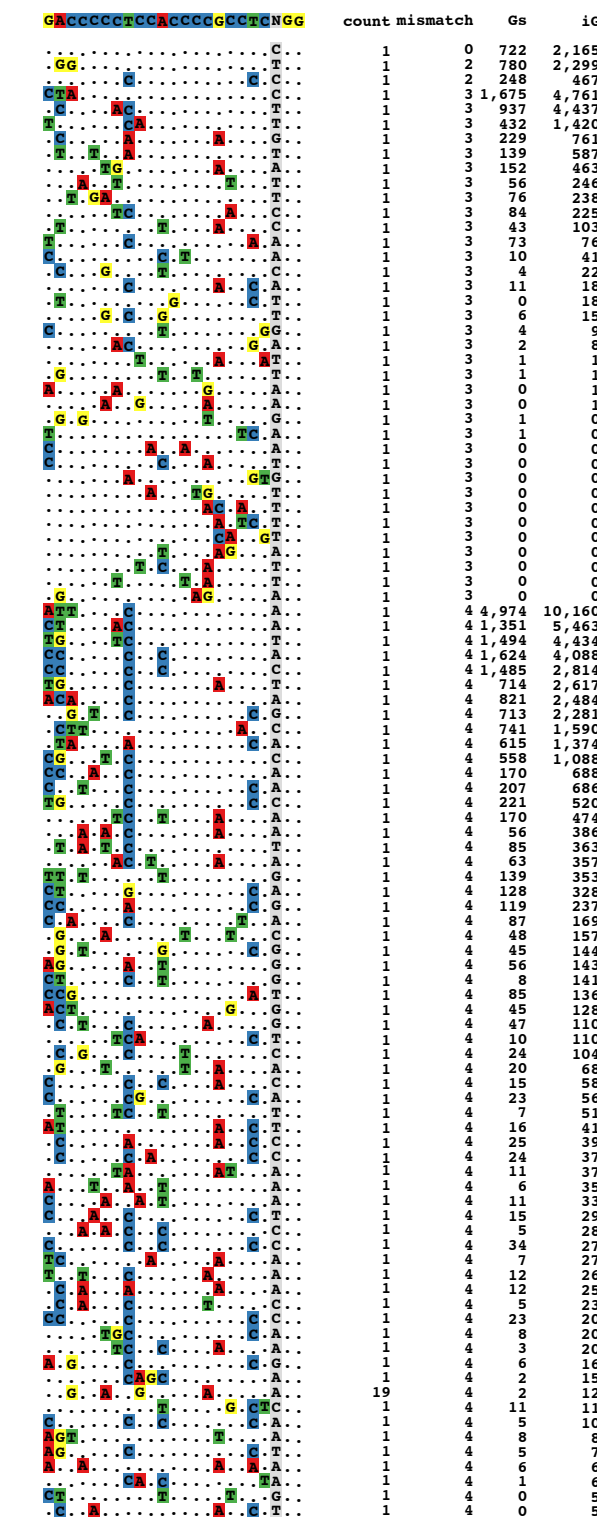D  
VEGFA(s3)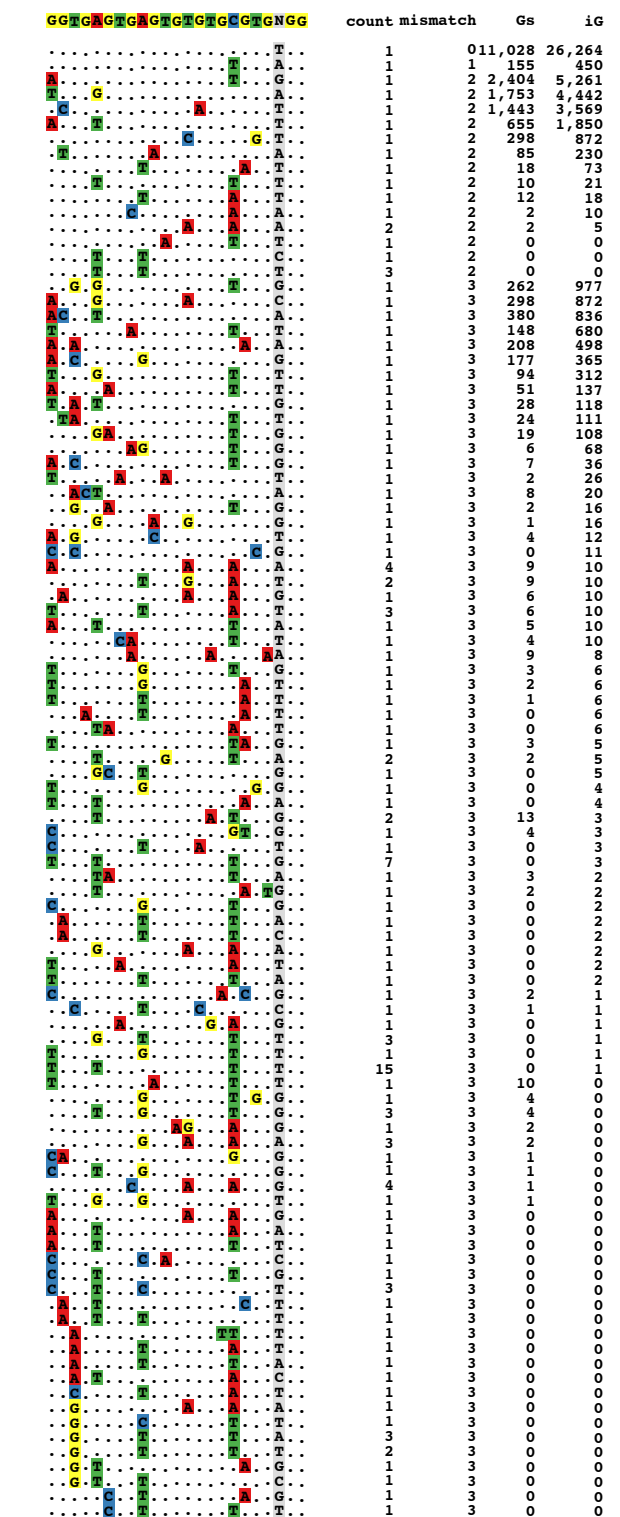

Supplement: Supplementary file 7 — Figure S2. Human genome sequences with near matches to the B2M, TRAC5, and VEGFA sgRNAs, with data on frequency of incorporation of the dsODN. “Count” indicates the frequency of the sequence in the human genome; “mismatch” indicates the number of mismatches relative to the sgRNA recognition sequence; “Gs” indicates the number of isolations from GUIDE-seq; and “iG” indicates the number of isolations from iGUIDE. (PDF 3508 kb) [file 13059_2019_1625_MOESM7_ESM.pdf]
