# Supplementary material for: iGUIDE: an improved pipeline for analyzing CRISPR cleavage specificity
Source: Genome Biol. 2019 Jan 17;20:14. doi: 10.1186/s13059-019-1625-3 (PMC6337799; doi:10.1186/s13059-019-1625-3)
Supplement: Supplementary file 5 — A Standard Operating Procedure for carrying out iGUIDE analysis. This report provides detailed protocols for carrying out the iGUIDE procedure. (PDF 107 kb) [file 13059_2019_1625_MOESM5_ESM.pdf]

iGUIDE protocol – Marking DSB through incorporation of DNA oligos

A. Construct double stranded oligo dinucleotide (dsODN)

1. Sequence of dsODN:
   Sense-strand:
/5Phos/G*C*TCGCGTTTAATTGAGTTGTCATATGTTAATAACGGTATACGC*G*A
   Antisense-strand:
/5Phos/T*C*GCGTATACCGTTATTAACATATGACAACTCAATTAAACGCGA*G*C

2. To anneal the strands of the dsODN, mix sense and anti-sense DNA at equimolar concentrations, heat to remove secondary structures and partially annealed oligos, then cool mixture slowly to allow for stringent binding to complementary sequences. The below thermocycler protocol can be used for the heating and slow cooling steps:

Table P1. Anneal dsODN strands

**Step**	**Time**	**Temperature (°C)**
Initial heating	1 min	95
Gradual Temp. Decrease	-Δ0.5°C per 1 min 30 sec	95 -> 4

3. Store annealed dsODN for extended periods of time at -20°C and try to avoid multiple freeze thaw cycles.

B. Transfect the dsODN with designer nuclease(s)

As the dsODN needs to be readily available to incorporate into double strand breaks, it will need to be present during genome editing and should be introduced along with other components.

1. For nucleases delivered by encoding mRNA, it is recommended to deliver 100 to 500 pmols of dsODN along with guide RNAs or along with the mRNA if guide molecules are not introduced.

2. For nucleases delivered as protein, it is recommended to deliver 100 to 500 pmols of dsODN along with the protein.

iGUIDE protocol – Sequencing Incorporation Sites

A. Anneal sample linkers

Linkers or Adapters used in this method were consistent with those proposed in Tsai, SQ *et al.* 2015.

1. Adapters are formed from annealing the adapter common oligo with the adapter oligos (A01 to A16, sequences present in iGUIDE Nucleotide Table). These adapters contain a random 8-mer sequence of NNWNNWNN where N can be A, T, G, or C and W can be A or T. The random sequence can be used as a unique molecular index (UMI) during data processing.

Table P2. Y-Adapter preparation reaction

Component	Volume (µL)	Conc. (µM)
TE Buffer	80.0	1X
Adapter Oligo A##	10.0	100
Adapter Common Oligo	10.0	100

2. Thermocycler protocol:

Table P3. Linker annealing protocol

Step	Time	Temperature (°C)
Initial heating	1 min	95
Gradual Temp. Decrease	-Δ0.5°C per 1 min 30 sec	95 -> 4

3. Store annealed adapters at -20°C.

Note: Various linker designs have been proposed by different studies. PCR primers, conditions, and sequencing protocols may change depending on the molecular design of the linker or adapter.

B. Prepare and quantify input DNA

1. Sample concentration of genomic DNA should be quantified by Quant-iT PicoGreen dsDNA Assay Kit (PicoGreen Kit). Samples are initially quantified using 1 µL of sample.

2. Purify genomic DNA using AMPure XP beads (Beckman Coulter) at a ratio to sample volume of 1.8. Measure sample concentration again by PicoGreen Kit to have an initial measurement on input quantities of DNA. Elute samples with 40 µL of 1x TE buffer.

C. Fragmenting DNA through ultrasonication

1. Randomly shear samples to a target size of 800 – 900 bp on a Covaris M220 unit using AFA microtubes. The parameters for shearing are as follows:

- Sample volume : 130 µL
- Buffer : 1x TE buffer
- Peak power : 50 Watts
- Duty factor : 5%
- Cycles / Burst : 200
- Treatment time : 60 seconds
- Water temperature : 25°C

2. Purify samples after sonication using AMPure XP beads, with a bead to sample volume ratio of 1.0, and eluted with 80 µL of 1X TE buffer for processing by the positive and negative sequencing method or 40 µL of 1X TE buffer for processing by one method only.

D. End repair and TA-ligation

1. Use 40 µL of purified sample for the end-repair reaction (NEBNext Ultra End Repair / dA Tailing Module, E7442L). Carry out reactions in 200 µL PCR tubes. Prepare the following formulation for each reaction:

Table P4. End Repair / dA-Tailing Formulation

Component	Volume (µL)
Purified DNA	40.0
Mol. Grade Water	15.5
10X End Repair Buffer	6.5
Enzyme	3.0
Total	65.0

2. Incubate prepared reactions in a thermocycler at 20 °C for 30 minutes followed by a 65 °C incubation for another 30 minutes. Cool samples to 4 °C prior to TA-ligation preparation.

3. Use different annealed linkers for each sample within the preparation. Prepare each sample with the following formulation:

Table P5. TA-Ligation Formulation

Component	Volume (µL)
End Prepped DNA reaction	65.0
Blunt/TA Ligase Master Mix	15.0
Annealed Linker/Adapter (20 µM)	4.0
Ligation Enhancer	1.0
Total	85.0

4. Incubate reactions at 16 °C for 16 hours and then hold at 4 °C till the reactions are collected.

5. Purify each ligation reaction using AMPure XP beads at a bead to sample volume ratio of 0.9 and eluted in 40 µL of 1x TE buffer.

E. PCR1 – first step of nested-PCR

1. Use the following formulation for PCR1. Switch GSP1 primer depending on sequencing method (positive or negative).

Table P6. PCR1 Formulation (Master Mix)

Component	Volume (µL)
Molecular Grade Water	47.6
Taq Polymerase Buffer (10x, Mg^{2+} free)	12.0
TMAC (0.5 M)	6.0
$MgCl_2$ (50 mM)	4.8
GSP1 Primer (10 µM) (Positive or Negative)	4.0*
Adapter PCR1 Primer (P5_1, 10 µM)	2.0**
dNTP mix (10 mM)	2.4
Platinum Taq Polymerase (5 units/µL)	1.2
Total	80.0

* GSP1 primers are associated with sequencing from the 5’ (positive) or 3’ (negative) end of the dsODN once incorporated. Only one primer is used per reaction.

2. Separate each reaction into four technical replicates of even volume (10 µL each). Mix each replicate with 20 µL of master mix.

3. Amplify the reactions using the following protocol:

Table P7. PCR1 Thermal Protocol

Cycles	Step	Time	Temperature (°C)
-	1	5 min	95
14	2	30 sec	95
	3	5 min	65* (20% ramping down from 95)
-	4	5 min	72
-	5	hold	4

F. PCR2 – second step of nested-PCR

1. Use the following formulation for PCR2. Switch GSP2 primer between sequencing methods (positive and negative):

Table P8. PCR2 Formulation (Master Mix)

Component	Volume (µL)
Molecular Grade Water	73.6
Taq Polymerase Buffer (10x, Mg^{2+} free)	12.0
TMAC (0.5 M)	6.0
$MgCl_2$ (50 mM)	4.8
GSP2 Primer (10 µM) (Positive or Negative)	4.0*
Adapter PCR2 Primer (P5_2, 10 µM)	2.0**
dNTP mix (10 mM)	2.4
Platinum Taq Polymerase (5 units/µL)	1.2
Total	106.0

2. Transfer sample DNA directly from PCR1 reactions to respective replicate PCR2 reactions. Mix each replicate as in Table P9. Use distinct adapter and barcoded primer combinations to distinguish technical replicates.

Table P9. Replicate-Specific formulation

Component	Volume (µL)
Master Mix	26.5
PCR1 Product	2.0
P7XX Barcode Primer (10 µM)	1.5
Total	30.0

3. Amplify the reactions prepared for PCR2 using the following protocol:

Table P10. PCR2 Thermal Protocol

Cycles	Step	Time	Temperature (°C)
-	1	5 min	95
14	2	30 sec	95
	3	5 min	65* (20% ramping down from 95)
-	4	5 min	72
-	5	hold	4

4. Pool technical replicates amongst samples and purify using AMPure XP beads at 0.7 volume ratio of beads to sample. Elute purified DNA in 30 µL of 1X TE buffer.

G. Library preparation and quantification

1. Quantify each sample by PicoGreen and Kapa qPCR assay to determine the total and sequence-specific concentration of amplified DNA.

2. Pool samples into a single library of equimolar concentration (or a reasonable distribution of sample molar concentrations) based on the outcome of the Kapa qPCR assay.

3. If a library concentration is estimated below optimal sequencing concentrations (limit 1.1 nM), then the library should be concentrated to 30 µL using AMPure XP beads at a ratio of 0.7. Should the library remain under the limit concentration, PCR2 should be repeated to generate more sample and the additional sample should be pooled and purified, consistent with previous steps.

4. Characterize library concentration and size distribution by Kapa qPCR and a BioAnalyzer (Agilent Technologies: 2200 TapeStation), respectively.

H. Library sequencing (300 cycle – MiSeq Reagent Kit)

1. Denature library according to suggested Illumina sequencing protocols by incubating a small portion (5 µL of 2 or 4 nM) of the library with 0.2 M NaOH for 5 minutes at room temperature (final volume 10 µL). Neutralize chemical denaturation reactions with 600 - 990 µL of supplied buffer and place sample on ice. Formulate the loading sample to consist of approximately 10 – 12 pM library and 1 – 2 pM phiX DNA within a 600 µL volume.

2. Supplement Index 1 and Read 2 sequencing primers into the reagent cartridge to capture sequence from the oligo portion of the amplicons. Add these two primers to reagent wells 13 and 14 (for MiSeq reagent cartridges), respectively, rather than the custom primer wells. Approximately 3.4 µL of 100 µM primer is sufficient for sequencing runs. Mix with reagent by removing contents of reagent well with a transfer pipet, supplement with corresponding primer, and vortex. Return mixed reagent to its corresponding well and sealed till use. Remove seal prior to loading in refrigerated compartment of instrument.

3. Cycling allocations should be adjusted to match Table P11 for MiSeq instruments. Adjusting the Read 1 and Read 2 cycles are typically straight forward when setting up a run-specific sample sheet. Index 1 and Index 2 sequence cycles depend on the length on input sequences for samples. Input TTTTTTTT for Index 1 and TTTTTTTTTTTTTTTT for Index 2 for a bogus sample and allow the iGUIDE software to demultiplex the input sequencing files.

Table P11. MiSeq Cycling Protocol

Sequencing Portion	Dedicated Cycles
Read 1	151
Index 1	8
Index 2	16
Read 2	151

4. Output files contain independent index read FASTQ files.

I. Analysis of sequence data

Analyze obtained sequence data using the iGUIDE pipeline, available at https://github.com/cnobles/iGUIDE. After installation, set up a run configuration file (containing run-specific parameters, i.e. sgRNA sequences) and a sample information file (containing sample-specific parameters, i.e. Barcodes) before processing. For additional information, please see the iGUIDE documentation.
