# Supplementary material for: iGUIDE: an improved pipeline for analyzing CRISPR cleavage specificity
Source: Genome Biol. 2019 Jan 17;20:14. doi: 10.1186/s13059-019-1625-3 (PMC6337799; doi:10.1186/s13059-019-1625-3)
Supplement: Supplementary file 6 — Automated iGUIDE Summary Report. This reproducible report includes specifics on the samples sequenced and annotated data for each, generated by a standardized software pipeline. (PDF 1035 kb) [file 13059_2019_1625_MOESM6_ESM.pdf]

iGUIDE Summary Report

report generated : 2018-11-16
software version : v0.2.4
environment build : v0.1.0

Contents

Summary **2**

Specimen overview **3**

On-target analysis **4**
Specimen breakdown 5
Editing near known genomic sites 5

Off-target analysis **7**
Specimen breakdown 7
Off-target enrichment in cancer-associated genes 8
Genomic distribution of incorporation sites 9
Off-target gRNA sequence comparison 11

This report includes data from the following sequencing runs: iGUIDE Set1, iGUIDE Set2, iGUIDE Set3, and iGUIDE Set8.

Summary

The following document summarizes the results of processing iGUIDE Set1, iGUIDE Set2, iGUIDE Set3, and iGUIDE Set8 sequencing set(s) through the iGUIDE pipeline. Included in this document are explanations of the data analytics as well as tables and graphics of the data obtained from the sequence analysis. This report includes 16 specimens treated with 4 guide RNAs. A total of 18,626,117 reads are considered in this analysis, which represent 476,405 inferred cells sampled.

Specimen overview

Table 1: Specimen summary.

specimen	RNP	gRNA	dsODN	Reads	UMItags	Alignments
iG06	Cas9	B2M	iG	1,589,790	160,103	28,538
iG07	Cas9	B2M	iG	1,208,094	90,823	17,870
iG08	Cas9	B2M	iG	1,666,816	103,375	23,750
iG09	Cas9	B2M	iG	1,342,608	92,095	18,776
iG10	Cas9	B2M	iG	1,142,739	60,698	15,365
iG16	Cas9	TRAC5	iG	1,469,857	96,048	21,997
iG17	Cas9	TRAC5	iG	563,759	44,027	12,780
iG18	Cas9	TRAC5	iG	894,124	87,299	18,762
iG19	Cas9	TRAC5	iG	1,202,450	77,287	23,240
iG20	Cas9	TRAC5	iG	1,128,364	107,017	21,344
iG48	Cas9	VEGFAs2	iG	985,533	96,680	48,290
iG49	Cas9	VEGFAs2	iG	1,337,602	145,269	59,910
iG50	Cas9	VEGFAs2	iG	1,877,187	199,180	82,053
iG54	Cas9	VEGFAs3	iG	685,907	59,987	26,506
iG55	Cas9	VEGFAs3	iG	1,068,461	109,833	37,754
iG56	Cas9	VEGFAs3	iG	462,826	44,266	19,470

Each specimen started in the iGUIDE pipeline as genomic DNA. The gDNA was randomly sheared through ultrasonication and ligated with barcoded DNA linkers. Nested-PCR was used to amplify from incorporated dsODN sequences to the linker sequences with barcoded and linker-specific primers. This dual barcoding reduces sample to sample crossover. Amplicons were sequenced on an Illumina platform and the sequencing data processed with the iGUIDE software, available on **GitHub@cnobles/iGUIDE**.

DNA sequence reads were aligned to the hg38 reference genome. The number of reads aligning for each specimen is displayed in **Table 1**, along with the number of unique alignments they represent (the inferred cells sampled). Multiple reads may represent a singular alignment of genomic DNA, inherent to sequence analysis of amplified DNA. These alignments indicate individual events of dsODN incorporation and clonal expansion.

Alternatively, random nucleotide sequences are included in the ligated linker sequences. These Unique Molecular Indeces (UMItags) can provide another method of abundance by counting the number of UMItags and breakpoint position combinations for each incorporation sites. This method of quantification has an increased dynamic range, yet can suffer from PCR artifacts leading to inflated abundances.

On-target analysis

Incorporation sites, or locations in the genome where the dsODN was detected, are expected to be in the proximity of RNA-guided nuclease targeted locations. The guide RNAs provided for these analyses and their On-target locations (loci) are shown in **Table 2**. The genomic locations are in a format where chromosome, orientation, and nucleotide position are delimited by a colon (":").

Table 2: Guide RNAs and associated information.

Guide	gRNA	PAM	Edit Locus
B2M	`GAGTAGCGCGAGCACAGCTA`	`NGG`	chr15:-:44711569
TRAC5	`TGTGCTAGACATGAGGTCTA`	`NGG`	chr14:+:22547664
VEGFAs2	`GACCCCCTCCACCCCGCCTC`	`NGG`	chr6:-:43770825
VEGFAs3	`GGTGAGTGAGTGTGTGCGTG`	`NGG`	chr6:+:43769733

Analysis of On-target associated incorporation sites (**Table 3**) produces several features that are helpful in On- and Off-target site characterization. These include the following:

- Alignment **Pileups**: unique alignments that overlap with each other or "pileup", suggesting a nearby location may be targeted for a double strand break (DSB). For this analyses, any group of 3 or more unique alignments were considered as a pileup cluster.
- Flanking **Paired** alignments: alignments can be found on either side of a DSB, and therefore identifying flanking alignments suggests a DSB could be found between the paired alignments. Flanking alignments were searched for in these data up to 200 bp from each other.
- gRNA **Matched** alignments: searching for the guide RNA sequence upstream of the incorporation site can be an indicator of guided nuclease activity. While this indicator may seem to be crucial, guide RNAs have been demonstrated to have a variety of behaviors when annealing to target DNA, not all of which can be easily searched for with a simple sequence alignment. Nucleotide sequence matching treated gRNA sequences were searched for up to 100 bp upstream of the incorporation sites and required to have no more than 6 mismatches.

Specimen specific tables with data relating to these criteria are found in **Table 3** for percent On-target editing and **Table 4** for identified Off-target loci.

Specimen breakdown

Table 3 displays the percent of cells sampled that were associated with On-target loci for **All** alignments. Further the percentages for **Pileups**, **Paired**, and **Matched** criteria are displayed in the following columns.

Table 3: Percent On-target.

Specimen	Treatment	Condition	All Align.	Align. Pileups	Flanking Pairs	gRNA Matched
iG06	B2M	Cas9 - B2M - iG	6.994	70.55	96.88	99.75
iG07	B2M	Cas9 - B2M - iG	7.224	69.86	98.09	99.77
iG08	B2M	Cas9 - B2M - iG	5.916	59.43	96.79	99.72
iG09	B2M	Cas9 - B2M - iG	7.936	73.22	97.88	99.87
iG10	B2M	Cas9 - B2M - iG	8.721	72.2	98.52	100
iG16	TRAC5	Cas9 - TRAC5 - iG	3.164	48.88	91.13	99.14
iG17	TRAC5	Cas9 - TRAC5 - iG	4.21	65.05	98.53	99.26
iG18	TRAC5	Cas9 - TRAC5 - iG	4.061	63.18	97.3	98.7
iG19	TRAC5	Cas9 - TRAC5 - iG	2.22	47.91	94.46	99.23
iG20	TRAC5	Cas9 - TRAC5 - iG	2.928	54.68	95.55	98.27
iG48	VEGFAs2	Cas9 - VEGFAs2 - iG	1.145	2.105	2.538	2.274
iG49	VEGFAs2	Cas9 - VEGFAs2 - iG	1.168	2.537	2.705	2.75
iG50	VEGFAs2	Cas9 - VEGFAs2 - iG	1.066	2.481	2.675	2.717
iG54	VEGFAs3	Cas9 - VEGFAs3 - iG	16.93	25.94	54.76	27.16
iG55	VEGFAs3	Cas9 - VEGFAs3 - iG	14.29	25.64	54.79	27.33
iG56	VEGFAs3	Cas9 - VEGFAs3 - iG	17.37	26.6	55.27	27.35

Editing near known genomic sites

Figure 1 displays the distribution of dsODN incorporations around on-target site(s). Incorporations in different orientations are shown on the positive (red) and negative (blue) y-axis. The percentage in the bottom right corner of each plot is an estimate of the number of incorporations associated with the on-target site (based on pileups) captured within the allowed window of 100 bps. These data can be used to fine tune the processing analyses, specifically the `upstreamDist` parameter which modifies the distance upstream of incorporation sites to search for nuclease edited sequences.

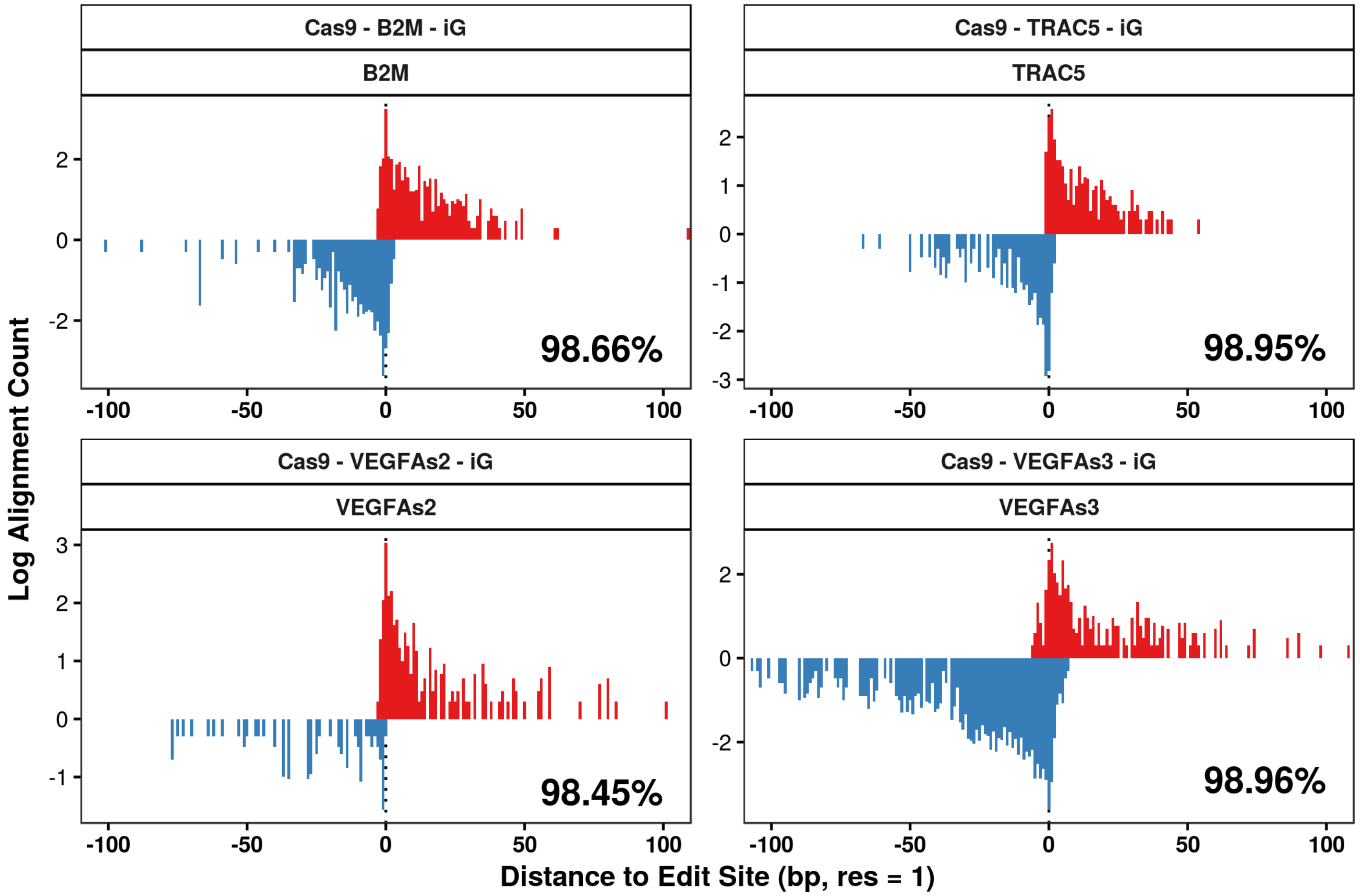

Figure 1: Distance distribution of observed incorporation sites from On-target loci.

Off-target analysis

Specimen breakdown

Using the criteria discussed previously based on characterizing features of nuclease targeted sites, off-target sites can be selected from the data in an unbiased manner. **Table 4** shows a summary of the unique off-target locations (loci) observed in the data. For **All** alignments, the loci are based on overlapping alignments (pileup cluster), without a minimum number of fragments required to be classified as a pileup cluster. **Pileup** loci are similarly based on overlapping alignments, but require at least 3 alignments to form a cluster. Flanking **Paired** loci require at least two unique alignments with opposite orientation (strands). Guide RNA **Matched** loci require a match in the upstream sequence to a treated gRNA (within 6 mismatches out of the 20 nts and zero PAM mismatches).

Table 4: Off-target Loci.

Specimen	Treatment	Condition	All Align.	Align. Pileups	Flanking Pairs	gRNA Matched
iG06	B2M	Cas9 - B2M - iG	24,636	223	27	4
iG07	B2M	Cas9 - B2M - iG	15,315	140	10	3
iG08	B2M	Cas9 - B2M - iG	20,391	234	20	4
iG09	B2M	Cas9 - B2M - iG	16,037	140	14	2
iG10	B2M	Cas9 - B2M - iG	12,957	128	9	0
iG16	TRAC5	Cas9 - TRAC5 - iG	19,687	188	30	6
iG17	TRAC5	Cas9 - TRAC5 - iG	11,554	75	4	4
iG18	TRAC5	Cas9 - TRAC5 - iG	16,908	119	10	8
iG19	TRAC5	Cas9 - TRAC5 - iG	21,438	138	14	4
iG20	TRAC5	Cas9 - TRAC5 - iG	19,491	130	12	10
iG48	VEGFAs2	Cas9 - VEGFAs2 - iG	21,898	628	262	379
iG49	VEGFAs2	Cas9 - VEGFAs2 - iG	31,918	676	283	381
iG50	VEGFAs2	Cas9 - VEGFAs2 - iG	45,998	795	377	430
iG54	VEGFAs3	Cas9 - VEGFAs3 - iG	9,045	255	55	136
iG55	VEGFAs3	Cas9 - VEGFAs3 - iG	16,389	378	74	181
iG56	VEGFAs3	Cas9 - VEGFAs3 - iG	6,620	169	42	122

Off-target enrichment in cancer-associated genes

Flanking **Paired** loci and gRNA **Matched** loci are tested for enrichment against specific gene lists in **Table 5**. The cancer-associated and special gene lists (adjusted in the config file) included in this analysis were: http://bushmanlab.org/assets/doc/allOnco_Feb2017.tsv and http://bushmanlab.org/assets/doc/humanLymph.tsv. Enrichment was tested by Fisher's Exact and p-values were adjusted for multiple comparisons using a Benjamani-Hochberg correction. Omitted specimens or conditions had insufficient data for this analysis (Total Gene Count = 0).

Table 5: Cancer-associated Gene editing enrichment.

Origin	Condition	Total Gene Count	Onco Related Count	Special Gene Count	Onco Enrich. p-value	Special Enrich. p-value
Reference	Random	2949	265	5	1.000	1.000
Flanking Pairs	Cas9 - B2M - iG	85	12	0	0.184	1.000
Flanking Pairs	Cas9 - TRAC5 - iG	75	10	0	0.280	1.000
Flanking Pairs	Cas9 - VEGFAs2 - iG	925	157	3	**0.000**	0.731
Flanking Pairs	Cas9 - VEGFAs3 - iG	174	31	5	**0.001**	**0.000**
gRNA Matched	Cas9 - B2M - iG	18	8	0	**0.000**	1.000
gRNA Matched	Cas9 - TRAC5 - iG	37	5	1	0.425	0.216
gRNA Matched	Cas9 - VEGFAs2 - iG	1193	214	4	**0.000**	0.653
gRNA Matched	Cas9 - VEGFAs3 - iG	442	122	30	**0.000**	**0.000**

Genomic distribution of incorporation sites

The figure(s) below display the genomic distribution of identified incorporation sites. The inner most ring plots all alignments identified within the associated data, while subsequent rings plot the alignments associated with Pileups, Flanking Pairs, and gRNA Matched groups. The height of the bar within its associated ring is correlated to the number of incorporations identified within the **10 Mb window** (normalized logarithm base 10 of incorporation sites).

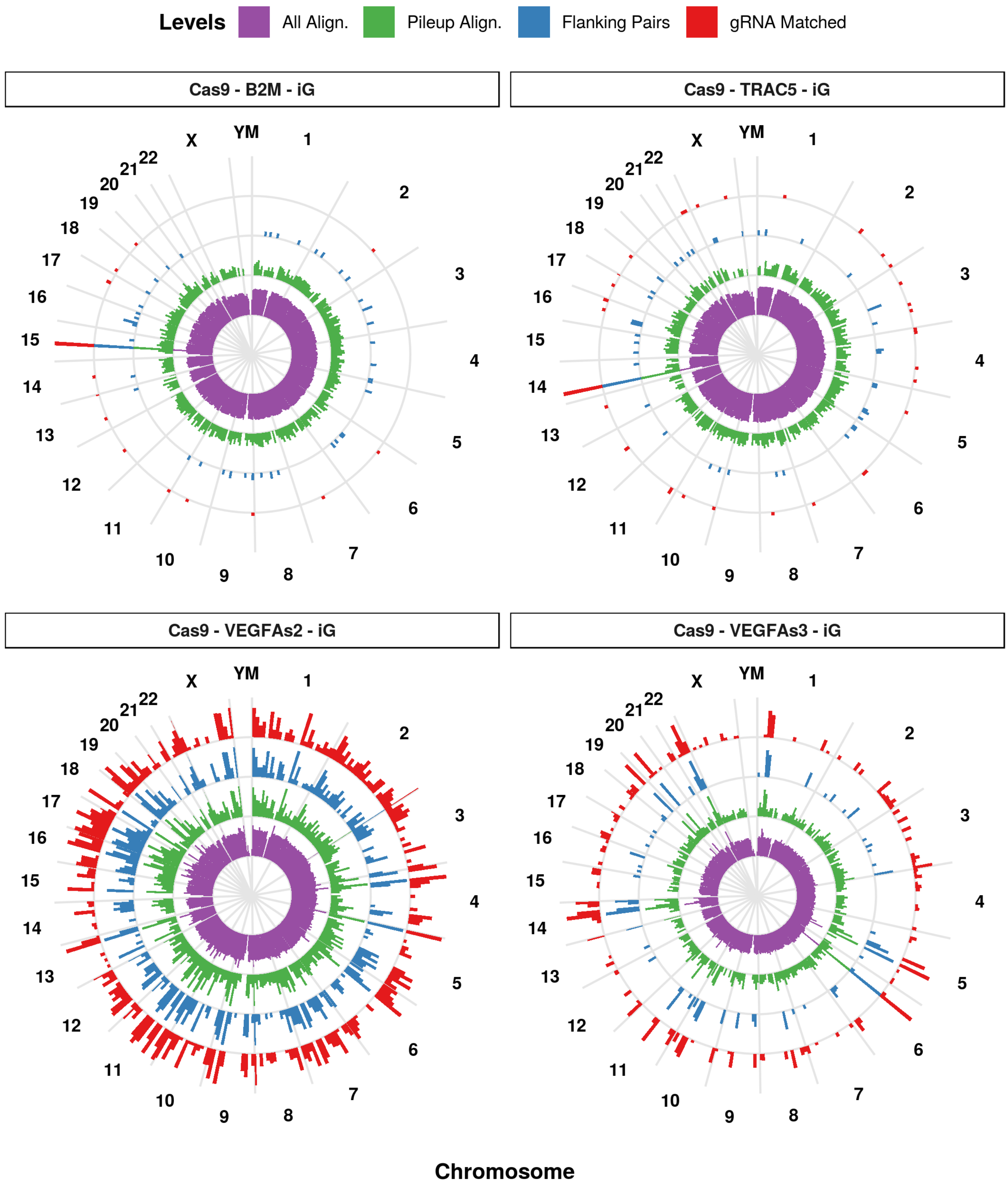

Figure 2: Genomic distribution of incorporation sites by bioinformatic characteristics.

Off-target gRNA sequence comparison

Off-target sites are identified by sequence similarity within 100 bp upstream of incorporation sites. The sequences of the gRNA matched sites are displayed below in Figure 3 along with the number of mismatches to the gRNA sequence (`mismatch`), an indication if the site is associated with an on- or off-target location (`target`), the total number of unique alignments associated with the site (`algns`), the maximum edit site likelyhood (`MESL`), and an identifier denoted by the nearest gene (`gene_id`). MESL is a score for the percentage likelyhood the off-target site is associated with directed nuclease editing, based solely on the respective On-target incorporation distribution. The gene name within the `gene_id` is the nearest gene to the genomic location. Further, symbols after the gene name indicate `*`) that the site is within the transcription unit of the gene, `~`) the gene appears on the cancer-association list, `!`) and that the gene appears on the special gene list. For this report, gene lists used were: http://bushmanlab.org/assets/doc/allOnco_Feb2017.tsv and http://bushmanlab.org/assets/doc/humanLymph.tsv.

Cas9 - B2M - iG
gRNA: B2M

GAGTAGCGCGAGCACAGCTANGG	mismatch	target	aligns	MESL	gene_id
....................A..	0	On	7,522	95.8	B2M*~
....GA..T......G.GG.G..	6	Off	2	21.8	CCDC182
AT.C..A.G...T.......G..	6	Off	1	81.9	GMFG
.T.G..A.G....G.....CT..	6	Off	1	25.0	MCF2L*
AG.....AG...T....A..T..	6	Off	1	21.7	PIEZO2*
...G..G...G..C...T.GT..	6	Off	1	21.6	RARG~
..A..T.TG.GA........A..	6	Off	1	21.4	STEAP2-AS1
...C..A...G...GT...GT..	6	Off	1	21.4	MUC5AC*~
C.C..T.A....A.....A.G..	6	Off	1	21.3	MTX2
......T.A.G..T...A.TT..	6	Off	1	20.6	LINC00856

Cas9 - TRAC5 - iG
gRNA: TRAC5

TGTGCTAGACATGAGGTCTANGG	mismatch	target	aligns	MESL	gene_id
....................T..	0	On	3,135	95.7	DAD1
...C.....G...T..C.CCA..	6	Off	2	31.5	CMIP*
.T.C.A.C......AC....G..	6	Off	2	26.7	AFF3~
AA..TG........A.....A..	5	Off	2	24.7	FAM19A2
A....C.TT...........G..	4	Off	1	95.7	JAKMIP1*~
.....A......AGA.AA..C..	6	Off	1	58.7	ADARB2~
.CA....A.T......G.C.T..	6	Off	1	40.8	LINC01173
G.....T..GC....A....A..	5	Off	1	36.0	LINC01179
.T......T...TTT....CA..	6	Off	1	34.8	C3orf58
..AA......T...A.....G..	4	Off	1	32.9	KIAA1210*

Cas9 - VEGFAs2 - iG
gRNA: VEGFAs2

GACCCCCTCCACCCCGCCTCNGG	mismatch	target	aligns	MESL	gene_id
ATT....C............A..	4	Off	10,281	95.3	HDLBP*~
CT....AC............A..	4	Off	5,533	95.3	LINC01258
CTA.................C..	3	Off	4,827	95.3	PAPD7*
TG....TC............T..	4	Off	4,484	95.3	ACLY*
.C....AC............T..	3	Off	4,465	95.3	LAMA3*
CC.....C..C.........A..	4	Off	4,163	95.3	CLYBL*
CC.....C..C.........C..	4	Off	2,883	95.3	SBF1*
TG.....C.......A....T..	4	Off	2,654	95.3	HERPUD1~
ACA....C............A..	4	Off	2,532	95.3	PLPPR1
.GG.................T..	2	Off	2,328	95.3	PAX6*~

Cas9 - VEGFAs3 - iG
gRNA: VEGFAs3

GGTGAGTGAGTGTGTGCGTGNGG	mismatch	target	aligns	MESL	gene_id
....................T..	0	On	13,261	96.5	VEGFA~
A...T...C.....G.GT..A..	6	Off	13,261	96.5	VEGFA~
A...............T...G..	2	Off	5,283	96.5	MAX*~
T...G...............A..	2	Off	4,480	96.5	COMMD10*
.C...........A......T..	2	Off	3,597	96.5	CYTH4
AC..T...............A..	3	Off	844	96.5	ZNF780B*
A.A..............A..A..	3	Off	508	96.5	LINC01339
A...T...............T..	2	Off	481	96.5	CFAP61*
T...T.A.T.....C.T...G..	6	Off	481	96.5	CFAP61*
A...T...C.....G.G.A.G..	6	Off	481	85.8	CFAP61*

Figure 3: Sequence similarity between off-target sites and targeting gRNA(s).
